# Exacerbation of ozone-induced pulmonary and systemic effects by β_2_-adrenergic and/or glucocorticoid receptor agonist/s

**DOI:** 10.1038/s41598-019-54269-w

**Published:** 2019-11-29

**Authors:** Andres R. Henriquez, Samantha J. Snow, Mette C. Schladweiler, Colette N. Miller, Janice A. Dye, Allen D. Ledbetter, Marie M. Hargrove, Judy E. Richards, Urmila P. Kodavanti

**Affiliations:** 1 Oak Ridge Institute for Science and Education, U.S. Department of Energy, Oak Ridge, Tennessee United States of America; 2 ICF, Durham, North Carolina United States of America; 3 Public Health and Integrated Toxicology Division, Center for Public Health and Environmental Assessment, U.S. Environmental Protection Agency, Research Triangle Park, North Carolina United States of America

**Keywords:** Toxicology, Environmental impact, Asthma

## Abstract

Agonists of β_2_ adrenergic receptors (β_2_AR) and glucocorticoid receptors (GR) are prescribed to treat pulmonary diseases. Since ozone effects are mediated through the activation of AR and GR, we hypothesized that the treatment of rats with relevant therapeutic doses of long acting β_2_AR agonist (LABA; clenbuterol; CLEN) and/or GR agonist (dexamethasone; DEX) would exacerbate ozone-induced pulmonary and systemic changes. In the first study, male 12-week-old Wistar-Kyoto rats were injected intraperitoneally with vehicle (saline), CLEN (0.004 or 0.02 mg/kg), or DEX (0.02 or 0.1 mg/kg). Since dual therapy is commonly used, in the second study, rats received either saline or combined CLEN + DEX (each at 0.005 or 0.02 mg/kg) one day prior to and on both days of exposure (air or 0.8ppm ozone, 4 hr/day x 2-days). In air-exposed rats CLEN, DEX or CLEN + DEX did not induce lung injury or inflammation, however DEX and CLEN + DEX decreased circulating lymphocytes, spleen and thymus weights, increased free fatty acids (FFA) and produced hyperglycemia and glucose intolerance. Ozone exposure of vehicle-treated rats increased bronchoalveolar lavage fluid protein, albumin, neutrophils, IL-6 and TNF-α. Ozone decreased circulating lymphocytes, increased FFA, and induced hypeerglycemia  and glucose intolerance. Drug treatment did not reverse ozone-induced ventillatory changes, however, lung effects (protein and albumin leakage, inflammation, and IL-6 increase) were exacerbated by CLEN and CLEN + DEX pre-treatment in a dose-dependent manner (CLEN > CLEN + DEX). Systemic effects induced by DEX and CLEN + DEX but not CLEN in air-exposed rats were analogous to and more pronounced than those induced by ozone. These data suggest that adverse air pollution effects might be exacerbated in people receiving LABA or LABA plus glucocorticoids.

## Introduction

Recently, we and others have shown that acute exposure to air pollutants, such as ozone, leads to a rapid release of stress hormones (i.e. epinephrine and cortisol/corticosterone) into the circulation through the activation of the neuroendocrine stress axes in rats^1–4^ and in humans^5^. We have further shown that systemic and pulmonary injury/inflammation effects of ozone are reduced in adrenalectomized rats with diminished circulating epinephrine and corticosterone^6^. When the receptors for epinephrine (β-adrenergic receptor, βAR) and corticosterone (glucocorticoid receptor, GR) were blocked by propranolol and mifepristone, respectively, ozone-induced systemic and pulmonary effects were inhibited in an antagonist-specific manner^7^. Specifically, ozone-induced vascular leakage was inhibited by each antagonist, but pulmonary neutrophilia was prevented only by βAR antagonist while lymphopenia was prevented by GR antagonist. Furthermore, when βAR and GR were activated by treating rats with high non-therapeutic doses of combined agonists, ozone-induced lung injury and inflammation were exacerbated^8^.

Agonists and antagonists of AR and GR are widely used for chronic illnesses involving cardiovascular, pulmonary and inflammatory conditions. Specifically, β_2_AR and/or GR agonists are commonly prescribed as singular or dual therapies for chronic lung diseases such as asthma and COPD^9^. Activation of β_2_AR, one of the many receptors of catecholamines located preferentially in the smooth muscle of the airways, induces relaxation in bronchoconstricted patients^10^. Activation of GR is associated with immunosuppression through downregulation of pro-inflammatory cytokine gene transcription, lymphocyte apoptosis, and migration of cells to/from lymphoid organs^11^. In the Salmeterol Multicenter Asthma Research Trial, death due to respiratory and asthma-related events was increased in patients taking a long acting β_2_AR agonist (LABA) monotherapy^12^. However, in a more recent study, LABA monotherapy was not associated with increased asthma-related events, and asthmatic patients taking LABA + inhaled corticosteroids experienced fewer asthma exacerbations than those using corticosteroids only^13^.

Given the role of AR and GR activation in acute air pollution-induced lung injury and inflammation^6–8,14^, and the use of AR and GR agonists in treatment of chronic asthma and COPD, it is conceivable that patients receiving bronchodilators and/or immunosuppressants might have differential inflammatory processes induced after acute air pollutant exposures^15–18^. For instance, it was found that asthmatic children living in Detroit have worsened responses to ozone and fine particulate matter when compared to those not receiving steroids^19^. Similarly, Gent and colleagues^20^ found that asthmatic children using maintenance medication were more vulnerable to ozone-induced pulmonary inflammation when compared to asthmatic children who did not receive medication. In addition, β_2_AR agonists were shown to exacerbate particulate matter-induced pulmonary inflammation and injury in mice through increased production of the pro-inflammatory cytokine IL-6^21^. More recently, Ritchie and collaborators^22^ demonstrated that β_2_AR agonists may promote lung inflammation through activation of the cyclic adenosine monophosphate response element in epithelial cells, while producing bronchodilator effects in smooth muscle cells. These studies suggest that single and/or dual therapies might exacerbate acute pulmonary injury/inflammation induced by air pollutants through activation of AR and GR. Our recent findings indicate that when rats were treated with relatively high non-therapeutic doses of LABA and corticosteroids given together (several folds higher than the therapeutic doses used for humans when adjusted to body weight), ozone-induced lung injury and inflammation were exacerbated in rats^8^. Thus, although we established the role of combined βAR and GR activation using higher (non-therapeutic) doses in ozone-induced pulmonary and systemic effects, the purpose of the current study was to examine if ozone-induced lung injury, inflammation, lymphopenia and metabolic alterations were exacerbated in rats receiving more therapeutically relevant dosages of LABA (clenbuterol; CLEN) or the GR agonist, dexamethasone (DEX), as separate treatments. Moreover, since dual therapy involving LABA and steroids are often recommended over single therapy, and because the dosages of dual drug treatment used in prior study were in a non-therapeutic range^8^, in the second study herein, we examined the interactive effects of ozone and the combination therapy of CLEN and DEX at therapeutically relevant doses.

## Materials and Methods

### Animals

Male Wistar-Kyoto rats (10–11 weeks of age) from Charles River Laboratory (Raleigh, NC) were pair housed in cages containing beta chip bedding in a room under controlled relative humidity (55–65%), temperature (21 °C), and dark/light cycle (12 h each). Rats were provided with water and standard Purina (5001) rat chow (Brentwood, MO) *ad libitum* unless stated. The animal facility is approved by an Association for Assessment and Accreditation of Laboratory Animal Care. Our animal protocols were approved by the U.S. Environmental Protection Agency’s Institutional Animal Care and Use Committee and all methods were performed in accordance with the relevant guidelines and regulations (NHEERL LAPR #19-05-002). We previously determined that 5–10% of male Wistar Kyoto rats develop spontaneous cardiac hypertrophy^23^ therefore, heart weights were recorded for all animals at necropsy. To avoid the influence of cardiac hypertrophy-related secondary pulmonary changes, those with a heart/body weight ratio above 20% of normal values were excluded from the study as we have reported in previous study^8^. This resulted in n = 6–8 for most endpoints.

### Drug treatments

**Figure 1:**
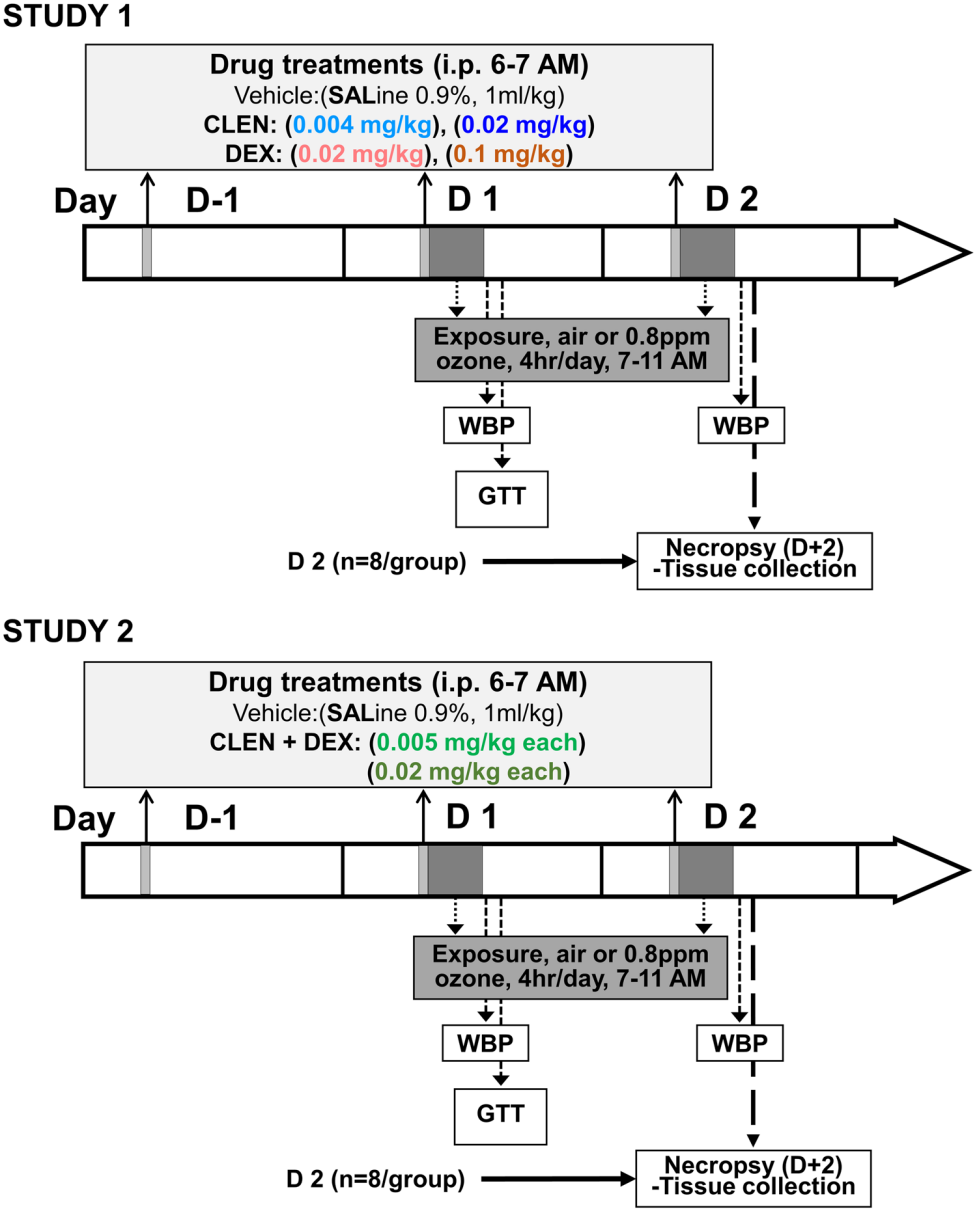
Schema of experimental design for study 1 and 2. The time line of drug treatments, exposures, and necropsies are indicated by arrows. Before air or ozone exposures began, rats were injected with vehicle (Saline, SAL), clenbuterol (CLEN), or dexamethasone (DEX) in study 1 and SAL or CLEN followed by DEX (CLEN + DEX) in study 2 at dose levels indicated. Injections were done in the mornings (~6–7 AM) daily starting one day prior to (D-1) and each day of exposure (D 1 and D 2). For both studies, animals were exposed to air or 0.8 ppm ozone (4 hr/day) for 2 consecutive days. Whole body plethysmography was performed within 30–45 min after each exposure day. Glucose tolerance testing was performed within 0.5–2.5 hr following first day of ozone exposure (D 1) after plethysmography, while necropsy and tissue collection were performed within 0.5–2.5 hr post second day of exposure (D 2) and plethysmography.

Experimental designs for both studies are detailed in Fig. 1. For each study, rats were randomized by body weight into their respective groups (n = 8/group). In study 1, rats were injected intraperitoneally with sterile saline (1 ml/kg; vehicle), clenbuterol hydrochloride (CLEN, Sigma-Aldrich, St Louis, MO) using a low [0.004 mg/kg] or high [0.02 mg/kg] dose, or dexamethasone sodium phosphate (DEX, Henry Schein, Dublin OH) as a low [0.02 mg/kg] or high [0.1 mg/kg] dose. In study 2, rats were injected intraperitoneally with either saline (1 ml/kg; vehicle), or CLEN followed by DEX (CLEN + DEX; within 5–10 minutes of each other) using a low (both at 0.005 mg/kg) or high (both at 0.02 mg/kg) dose. For each study, daily vehicle/drug treatment began one day before starting air or ozone exposure and continued each day of exposure for a total of 3 daily injections. The treatments occurred in the morning at ~06:30 AM followed by air or 0.8 ppm ozone exposures at ~07:00 AM (4 hr/day for two consecutive days).

Although CLEN is not currently prescribed for humans as a bronchodilator in the U.S., it is used as a performance-enhancing drug and weight loss supplement in humans^24,25^. As we have explained in previous study^8^, the recommended bronchodilation dose of CLEN is 0.02–0.06 mg/day for an average 70 kg human, however up to 0.12 mg/day doses (~0.0017 mg/kg body weight) are used for inducing weight loss^26^. DEX is widely prescribed in human and veterinary clinical practices and used in research. The recommended adult dose of DEX ranges from 0.75 to 9 mg which is taken every 6–12 hr for an average 70 kg human. At this level, DEX is anti-inflammatory. However, approximately 0.15 mg/kg/day doses are recommended for adrenal insufficiency^27^. Thus, for these studies, in order to examine potential interaction with ozone, we used these drugs at near therapeutic levels as indicated in Fig. 1, that did not produce lung injury or inflammation in air-exposed animals. However, after determining the influence of the single drug treatment in study 1, we noted that both the low and high doses of DEX resulted in marked lymphopenia (immunosuppressive effect) while CLEN did not influence any of the parameters assessed. Therefore, for the combination treatment in study 2, we further lowered the DEX doses (Fig. 1). We postulated that this regimen would be appropriate for examining the susceptibility of individuals receiving singular or combinational LABA and corticosteroid therapies to air pollution effects.

### Ozone exposures

Rats were exposed whole body to filtered air or 0.8 ppm ozone, 4 hr/day (~7–11 AM) for two consecutive days (Fig. 1)^8^. This concentration of ozone was higher than what may occur environmentally. However, rats have faster lung clearance mechanisms and varied lung deposition patterns compared to humans and thus are likely to require higher doses to achieve the same level of inflammatory insult^28^. Human clinical studies use 0.2 or even 0.3 ppm ozone during intermittent exercise^5^. In resting rats, the deposited ozone dose at 0.8 ppm is comparable to 0.2 ppm ozone in exercising humans^29^. Two-day exposure regimen allowed us to assess glucose tolerance immediately following day 1 exposure; and lung injury and inflammation immediately following day 2 exposure since lung effects are expected to be maximal by the second day^30^. Ozone was generated, delivered to the chamber and monitored as previously described^8^. For study 1, the ozone chamber concentration was 0.797 ± 0.010 ppm (mean ± SD). RH was 50.1 ± 0.4 and 46.2 ± 0.4%, temperature was 72.2 ± 0.2 and 73.6 ± 0.1 °F, and air flow was 260.5 ± 0.2 and 256 ± 0.1 L/min for filtered air and ozone chambers, respectively. For study 2, ozone concentration was 0.80 ± 0.002 ppm. RH was 48.0 ± 1.1 and 45.7 ± 1%, temperature was 73.0 ± 0.1 and 73.4 ± 0.01 °F, and air flow was 253.5 ± 0.3 and 255.9 ± 0.3 L/min for filtered air and ozone chambers, respectively.

### Whole Body Plethysmography

To evaluate possible drug-induced alterations in ventilation that could lead to changes in ozone dosimetry, rats were monitored using whole body plethysmography immediately after air or ozone exposure on D 1 and D 2. Rats were transported to a separate quiet room and placed in plethysmography chambers (n = 8 rats/run). They were allowed a 2 min acclimation period. Data were collected for an additional 5 min similar to our previous study^8^. In brief, spontaneous breathing parameters included (breathing frequency [*f*], tidal volume [TV], minute ventilation [MV], peak inspiratory flow [PIF], peak expiratory flow [PEF], and enhanced pause [Penh]). Penh is a composite parameter computed from the waveform of the box pressure signal during inspiration and expiration with the consideration of early and late expiration timings (Pause), and is generally indicative of labored breathing or bronchoconstriction^31^. All data were collected using EMKA iox 2 software and analyzed (SCIREQ, Montreal, Canada).

### Glucose tolerance test

Since glucose intolerance is one of the common systemic responses induced by the activation of neuroendocrine system, glucose tolerance testing was performed in fasted rats immediately after plethysmography as previously described^6^.

### Necropsy and tissue samples collection

Rats were necropsied within 0.5–2.5 hr after the second exposure (D 2)following euthanesia using Fatal Plus (sodium pentobarbital, Virbac AH, Inc., Fort Worth, TX; >200 mg/kg, intraperitoneal). Blood samples from the abdominal aorta were collected in vacutainer serum separator tubes and EDTA tubes^8^. A complete blood count was performed on a Beckman-Coulter AcT blood analyzer (Beckman-Coulter Inc., Fullerton, California). Thymus and spleens were weighed.

The right lung was lavaged and bronchoalveolar lavage fluid (BALF) total cell counts were performed using Z1 Coulter Counter (Coulter, Inc., Miami, FL)^8^. Cytospin slides were stained and cell differentials were performed (300 cells/slide) as previously described^8^. Total protein, albumin, and *N*-acetyl-β-D-glucosaminidase (NAG) activity were measured in the cell-free BALF using a Konelab Arena 30 clinical analyzer (Thermo Chemical Lab Systems, Espoo, Finland) as described^8^.

### Plasma and serum analysis

Epinephrine (adrenaline) and corticosterone plasma levels were measured using kits from Rocky Mountain Diagnostics (Colorado Springs, CO) and Arbor Assays (Ann Arbor, MI), respectively. Serum free fatty acids were measured using kits from Cell Biolabs, Inc (San Diego, CA). Free fatty acids (total) protocol was adapted for use on a Konelab Arena 30 clinical analyzer (Thermo Chemical Lab Systems, Espoo, Finland).

### Cytokine quantification

The concentrations of BALF cytokines (IL-6 and TNF-α) were determined using a V-plex custom rat cytokine panel (Mesoscale Discovery Inc., Rockville, MD) and following manufacturer’s protocol. To increase the sensitivity for detection of cytokines, sample volume per well was increased from the recommended 50 μL to 150 μL. In BALF samples from air control animals, those with levels of TNF-α below the assay detection limit, the values were imputed using the lowest quantified value in the group to avoid issues with the statistical analysis.

### Statistics

For all endpoints, each drug treatment (CLEN or DEX, study 1; or CLEN + DEX, study 2) was independently analyzed. Two-way analysis of variance (ANOVA) was used to analyze the effect of drug treatment and/or ozone exposure. The two independent variables for each study were exposure and treatment. For some endpoints, if normal distribution and homoscedasticity were not achieved, data were log transformed using Shapiro-Wilk and Levene’s tests, respectively. Area under the curve estimates for glucose tolerance test were calculated using the trapezoidal method. The correction for all multiple comparisons was done using Holm-Sidak *post-hoc* test and when a p-value of ≤0.05 was achieved, the differences were considered significant. The data (mean ± SEM) depicted in graphs and tables represent n = 6–8 animals/group. GraphPad Prism 7 (version 7.04) and Statext 2.7 software were used for statistical analysis and graph generation.

## Results

### Ozone exposure and drug treatments did not alter body weight but reduced thymic and splenic weights, and induced lymphopenia

**Figure 2:**
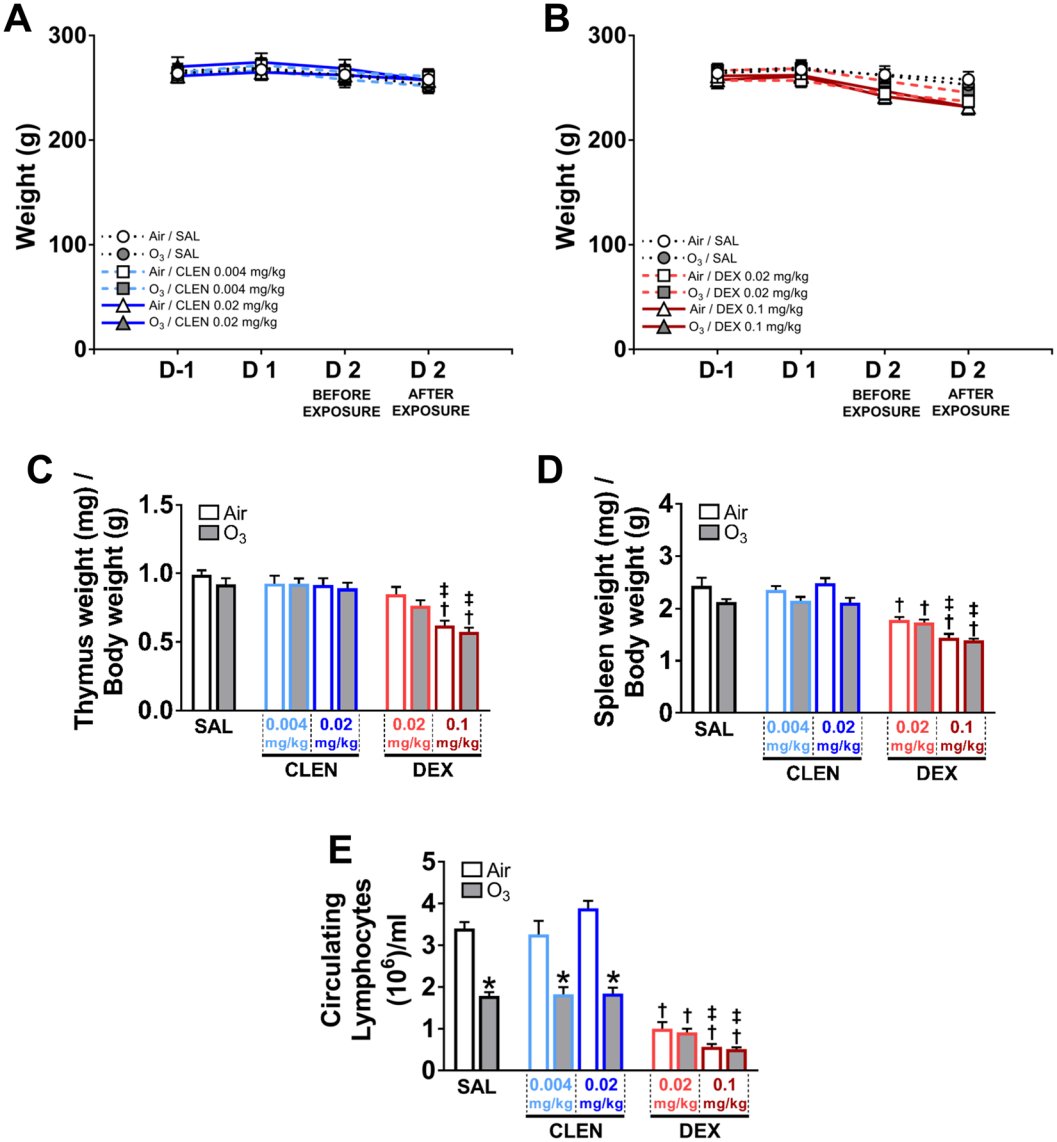
Ozone and/or CLEN or DEX treatments did not alter body weights, however DEX treatment reduced thymic and spleen weights, while both ozone and DEX reduced circulating lymphocytes (study 1). Body weights were assessed in rats at one day prior to (D-1), D 1, D 2 before exposure, and at D 2 immediately after exposure in CLEN (**A**) and DEX treatment groups (**B**). Thymus (**C**) and spleen (**D**) weights, and circulating lymphocytes (**E**) were assessed in rats during necropsy in all groups. Line and bar graphs show mean ± SEM of n = 6–8 animals/group. Significant differences between groups (p value ≤ 0.05) are indicated by * for ozone effect when compared to corresponding air-exposed rats, † for drug effect when compared to corresponding vehicle (SAL; saline)-treated rats, and ‡ for drug dose effect for the same exposure.

In study 1, body weights were not significantly changed by ozone exposure or by treatment with CLEN (Fig. 2A) or DEX (Fig. 2B). Thymic and splenic weights were assessed to characterize any immunosuppressive effects of the drug treatments or of the exposures. Ozone alone did not significantly reduce thymic and splenic weights in SAL-treated rats (Fig. 2C,D). However, the treatment with DEX but not CLEN significantly decreased thymus and spleen weights in a dose-dependent manner in both air and ozone-exposed rats when compared to SAL (Fig. 2C). Spleen weights were significantly decreased in ozone-exposed rats after high dose CLEN (Fig. 2D).

Ozone-induced lymphopenia has been reported in our previous studies^6,7^. In study 1, treatment with CLEN did not alter the number of circulating lymphocytes in air-exposed rats, however treatment with DEX at both dosages significantly depleted circulating lymphocytes in air-exposed rats (Fig. 2E). Ozone exposure also significantly depleted circulating lymphocytes in SAL and CLEN-treated rats, however, and due to the robust dose-dependent DEX effect on lymphocyte depletion, and additional ozone-induced lymphopenia in DEX-treated animals was not detactable (Fig. 2E).

**Figure 3:**
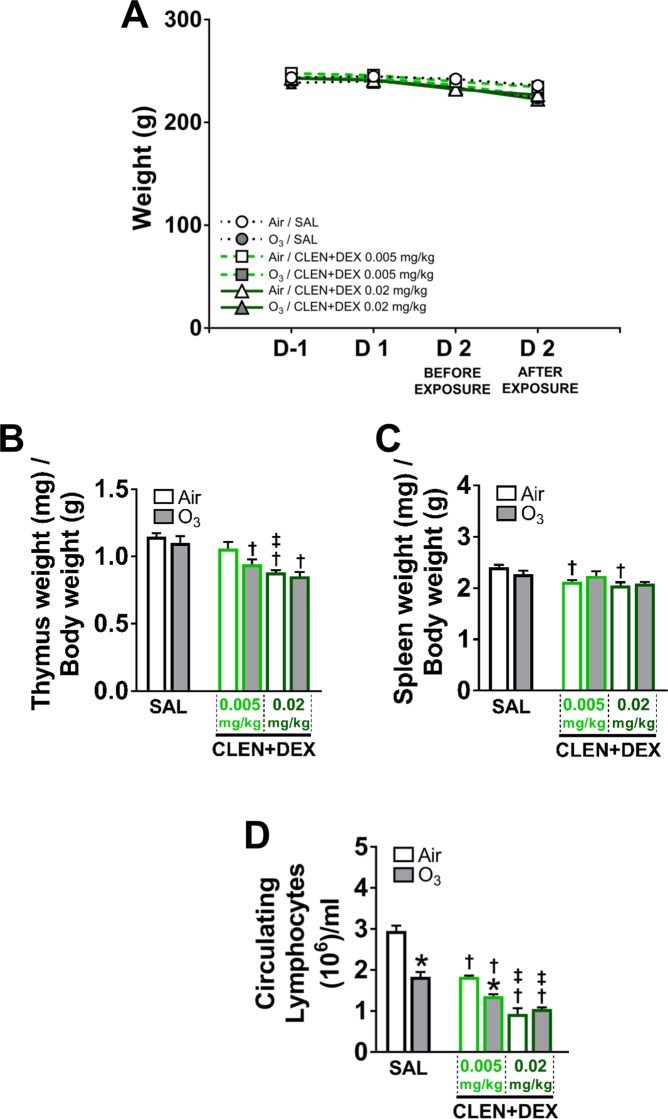
Ozone and/or CLEN + DEX treatments did not alter body weights; CLEN + DEX treatment reduced thymus and spleen weights, while both ozone and CLEN + DEX decreased circulating lymphocytes (study 2). Body weights (**A**) were assessed in rats at one day prior to (D-1), D 1, D 2 before exposure, and at D 2 immediately after exposure. Thymus (**B**) and spleen (**C**) weights, and circulating lymphocytes (**D**) were assessed in rats during necropsy. Bar graphs show mean ± SEM of n = 6–8 animals/group. Significant differences between groups (p value ≤ 0.05) are indicated by * for ozone effect when compared to corresponding air-exposed rats, † for drug effect when compared to corresponding vehicle (SAL; saline)-treated rats, and ‡ for drug dose effect for the same exposure.

In study 2, neither the CLEN + DEX treatment nor ozone exposure significantly affected body weights (Fig. 3A). However, CLEN + DEX did decrease thymus weight in both air and ozone-exposed rats (Fig. 3B) and spleen weights in ozone-exposed rats when compared to saline-treated rats (Fig. 3C).

In study 2, treatment with CLEN + DEX at both dosages significantly depleted circulating lymphocytes in air-exposed rats (Fig. 3D). Ozone-induced depletion of circulating lymphocytes in CLEN + DEX-treated rats was not detectable at high dose due to the robust effect of this drug treatment (Fig. 3D).

### Ozone-induced changes in ventilation were not reversed by concurrent drug treatments

**Figure 4:**
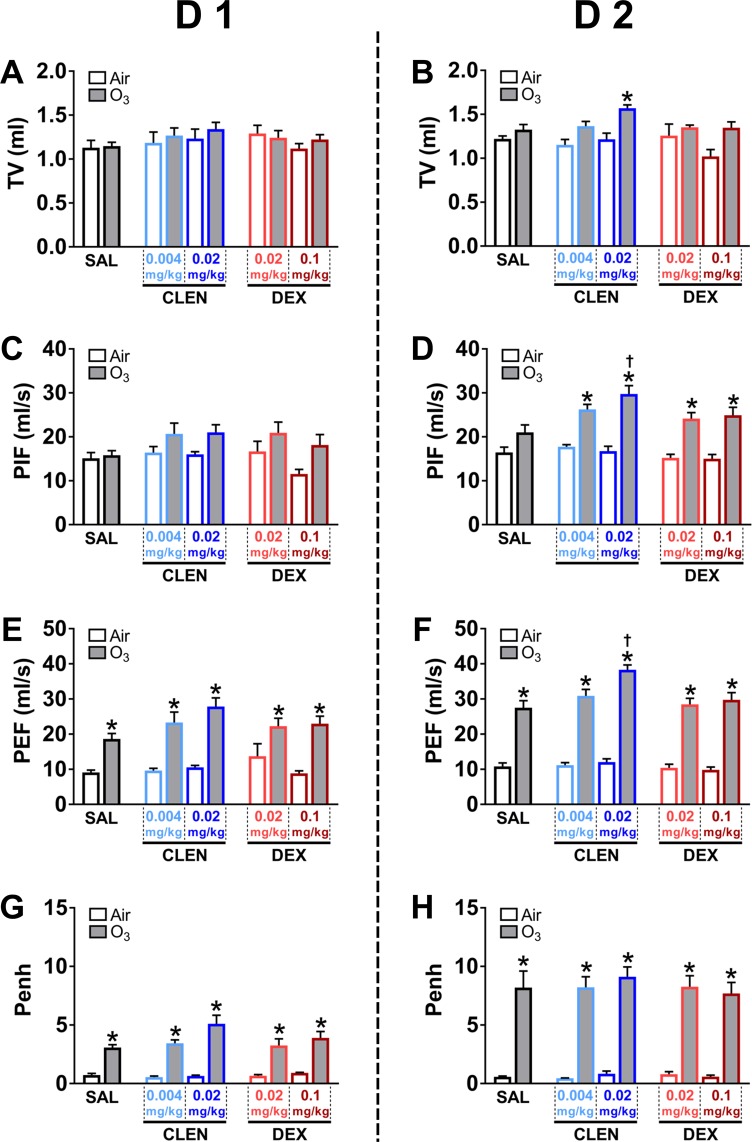
The influence of CLEN or DEX treatments on ozone-induced changes in ventilatory parameters (study 1). Of all ventilatory parameters assessed in rats through whole body plethysmography immediately after each day of air or ozone (0.8 ppm) exposure, the data for tidal volume (TV, **A,B**), peak inspiratory flow (PIF, **C,D**), peak expiratory flow (PEF, **E,F**), and enhanced pause (Penh, **G,H**) are shown. Bar graphs show mean ± SEM of n = 6–8 animals/group. Significant differences between groups (p value ≤ 0.05) are indicated by * for ozone effect when compared to corresponding air-exposed rats, and † for drug effect when compared to corresponding vehicle (SAL; saline)-treated rats.

**Figure 5:**
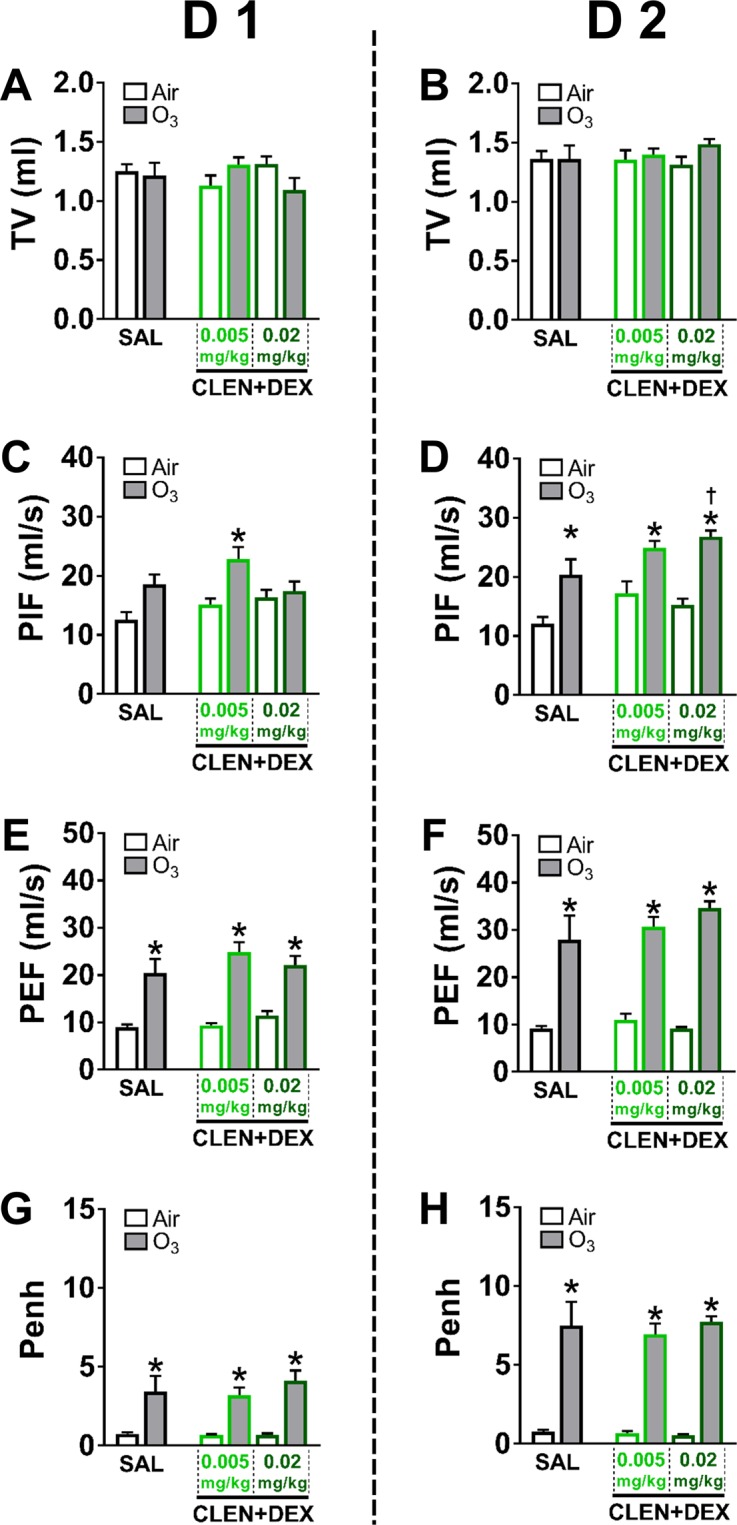
The influence of CLEN + DEX treatments on ozone-induced changes in ventilatory parameters (study 2). Of all ventilatory parameters assessed in rats through whole body plethysmography immediately after each day of air or ozone (0.8 ppm) exposure, the data for tidal volume (TV, **A,B**), peak inspiratory flow (PIF, **C,D**), peak expiratory flow (PEF, **E,F**), and enhanced pause (Penh, **G,H**) are shown. Bar graphs show mean ± SEM of n = 6–8 animals/group. Significant differences between groups (p value ≤ 0.05) are indicated by * for ozone effect when compared to corresponding air-exposed rats, and † for drug effect when compared to corresponding vehicle (SAL; saline)-treated rats.

To understand the effects of drug treatment on breathing and the potential to influence inhaled ozone dosimetry, whole body plethysmography was performed immediately after each day of air or ozone exposure. No consistent changes were observed in *f* or MV calculated in rats due to drug treatment or exposure conditions in either study (data not shown). In study 1, CLEN or DEX treatment alone did not significantly alter ventilatory parameters in air-exposed rats on D 1 or D 2 (Fig. 4). After ozone exposure, TV was not significantly changed, except for an increase in the high dose CLEN treatment group on D 2 (Fig. 4A,B). In general, ozone exposure was associated with significantly increased PIF (mainly at D 2, Fig. 4C,D), PEF and Penh (on both D 1 and D 2) (Fig. 4E,H). For a comparable breathing frequency, such changes are consistent with increased ventilatory effort. At D 2, rats receiving high dose CLEN treatment had exacerbated ozone-induced increases in both PIF and PEF (Fig. 4D,F). The magnitude of these changes was greater by D 2, consistent with worsening of respiratory distress (i.e., labored breathing and/or bronchoconstriction). The high dose CLEN group had proportionate increases in PIF and PEF which when combined with greater TV, suggests development of both expiratory effort (i.e. bronchoconstriction) and hyperpnea (i.e., increased TV with no corresponding decrease in breathing frequency). Such changes suggest development of a combination of pulmonary edema and airflow obstruction. For study 2, similar to study 1, CLEN + DEX treatment did not influence breathing parameters in air controls. However, ozone exposure again was associated with increased PIF (on D 2; Fig. 5C,D), PEF and Penh (on both D 1 and D 2) (Fig. 5E–H). Importantly, ozone-induced changes in Penh were not reversed by CLEN + DEX treatment on D 1 or D 2 (Fig. 5G,H).

### Ozone-induced lung protein leakage and inflammation were exacerbated by CLEN treatment

**Figure 6:**
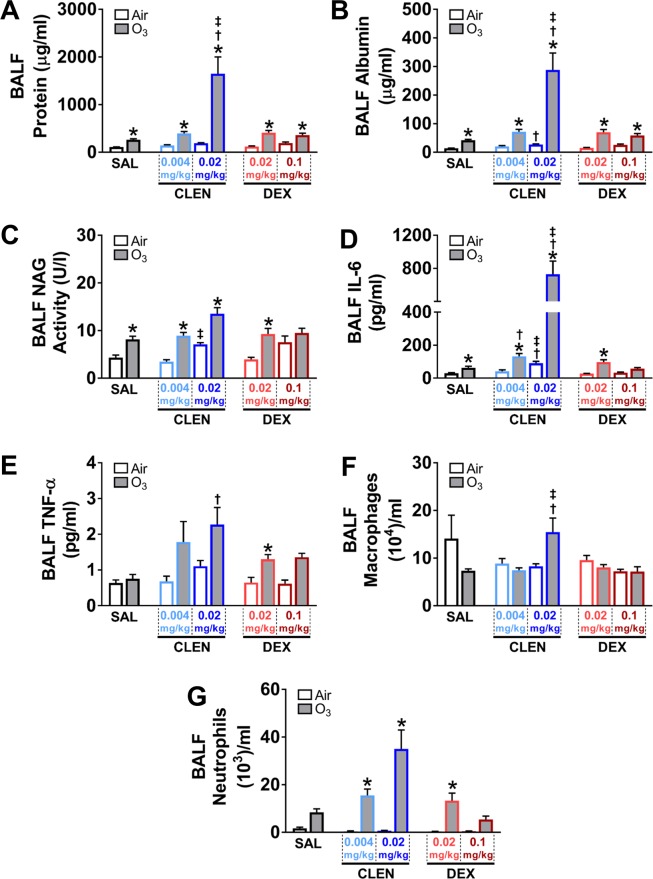
CLEN but not DEX treatment exacerbated ozone-induced lung protein leakage, cytokine changes and neutrophilic inflammation (study 1). BALF protein (**A**), albumin (**B**), NAG activity (**C**), IL-6 (**D**), TNF-α (**E**), macrophage numbers (**F**), and neutrophil numbers (**G**) were assessed in rats after the second day of air or ozone (0.8 ppm) exposure (4 hr/day for 2 consecutive days). Bar graphs show mean ± SEM of n = 6–8 animals/group. Significant differences between groups (p value ≤ 0.05) are indicated by * for ozone effect when compared to corresponding air-exposed rats, † for drug effect when compared to corresponding vehicle (SAL; saline)-treated rats, and ‡ for drug dose effect for the same exposure.

BALF protein and albumin were assessed to quantify pulmonary microvascular leakage, while BALF pro-inflammatory cytokines, macrophage and neutrophil count were quantified to assess pulmonary inflammation. In study 1, neither CLEN nor DEX treatment increased BALF protein or albumin leakage in air-exposed rats except for the high dose CLEN which modestly increased albumin levels. Ozone-induced increases in BALF protein and albumin were apparent in SAL-treated rats (Fig. 6A,B). CLEN, but not DEX, at the high dose exacerbated ozone-induced pulmonary protein and albumin leakage (Fig. 6A,B). CLEN or DEX treatment did not change BALF NAG activity (a marker of macrophage activation) in air-exposed rats except for a small increase in animals treated with high dose of CLEN. Ozone exposure was associated with increases in BALF NAG activity in SAL-treated rats. This ozone effect on NAG activity was not significantly affected by CLEN or DEX (Fig. 6C). Pro-inflammatory cytokines IL-6 and TNF-α were quantified in BALF to determine the degree and severity of the pulmonary inflammatory response. In air-exposed rats, only high dose CLEN treatment increased BALF IL-6 (Fig. 6D). IL-6 was increased after ozone exposure in SAL treated rats. CLEN but not DEX markedly exacerbated ozone-induced IL-6 increases in a dose-dependent manner (Fig. 6D). BALF TNF-α levels were not as consistent as IL-6 and high variability was noted in the data. Neither CLEN nor DEX treatments changed BALF TNF-α levels in air-exposed rats. TNF-α levels were slightly increased by ozone in most treatment groups; however, levels were only significantly increased in animals given high dose CLEN or low dose DEX (Fig. 6E). In air-exposed rats, there were no effects of either drug treatment on BALF macrophage numbers. BALF macrophages increased in those treated with high dose CLEN and exposed to ozone when compared to SAL (Fig. 6F). Ozone-induced pulmonary inflammation in rats was characterized by the recruitment of neutrophils to the alveolar space. CLEN or DEX did not increase neutrophils in air-exposed rats, however, ozone induced-neutrophilia was apparent in all rats. CLEN but not DEX treatment exacerbated ozone-induced neutrophilia in a dose-dependent manner (Fig. 6G).

**Figure 7:**
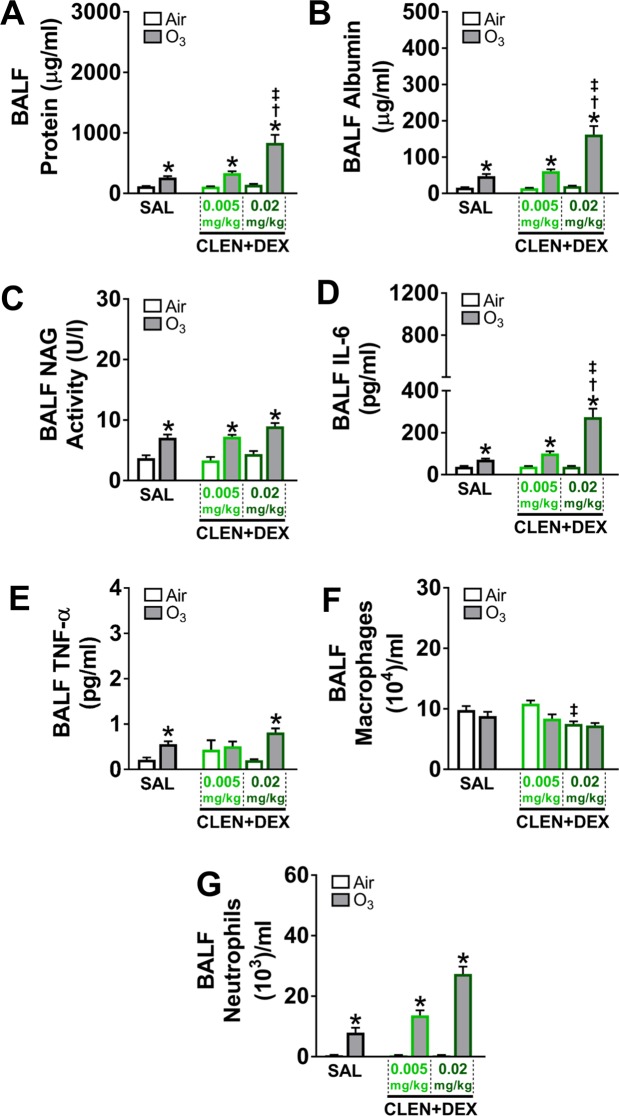
CLEN + DEX treatment exacerbated ozone-induced lung protein leakage, cytokine changes and neutrophilic inflammation (study 2). BALF protein (**A**), albumin (**B**), NAG activity (**C**), IL-6 (**D**), TNF-α (**E**), macrophage numbers (**F**), and neutrophil numbers (**G**) were assessed in rats after the second day of air or ozone (0.8 ppm) exposure (4 hr/day for 2 consecutive days). Bar graphs show mean ± SEM of n = 6–8 animals/group. Significant differences between groups (p value ≤ 0.05) are indicated by * for ozone effect when compared to corresponding air-exposed rats, † for drug effect when compared to corresponding vehicle (SAL; saline)-treated rats, and ‡ for drug dose effect for the same exposure.

In study 2, CLEN + DEX did not increase BALF protein and albumin leakage in air-exposed rats (Fig. 7A,B). Ozone increased BALF protein and albumin in SAL-treated rats. High dose CLEN + DEX when compared to SAL potentiated ozone-induced vascular protein and albumin leakage (Fig. 7A,B). CLEN + DEX treatments did not change BALF NAG activity in air-exposed rats but ozone exposure increased BALF NAG activity similarly in SAL and CLEN + DEX treated rats (Fig. 7C). CLEN + DEX treatment did not change BALF IL-6 in air-exposed rats (Fig. 7D). BALF IL-6 was increased after ozone exposure in SAL and CLEN + DEX-treated rats. High dose CLEN + DEX exacerbated ozone-induced IL-6 increase when compared with SAL treated-animals (Fig. 7D). BALF TNF-α did not change after CLEN + DEX treatment in air-exposed rats, however there was a small increase after ozone exposure in SAL and high dose CLEN + DEX-treated rats (Fig. 7E). BALF macrophage count was generally unaffected by CLEN + DEX or ozone exposure, except for a small decrease in air-exposed CLEN + DEX treated rats (Fig. 7F). BALF neutrophil numbers did not change by CLEN + DEX treatment in air-exposed rats however, ozone-induced neutrophilia was evident in all groups. Although not significant, the neutrophil count was higher in high dose CLEN + DEX-treated animals exposed to ozone when compared to SAL-treated air-exposed rats (Fig. 7G).

### Ozone-induced metabolic changes were exacerbated by DEX and CLEN + DEX treatments

**Figure 8:**
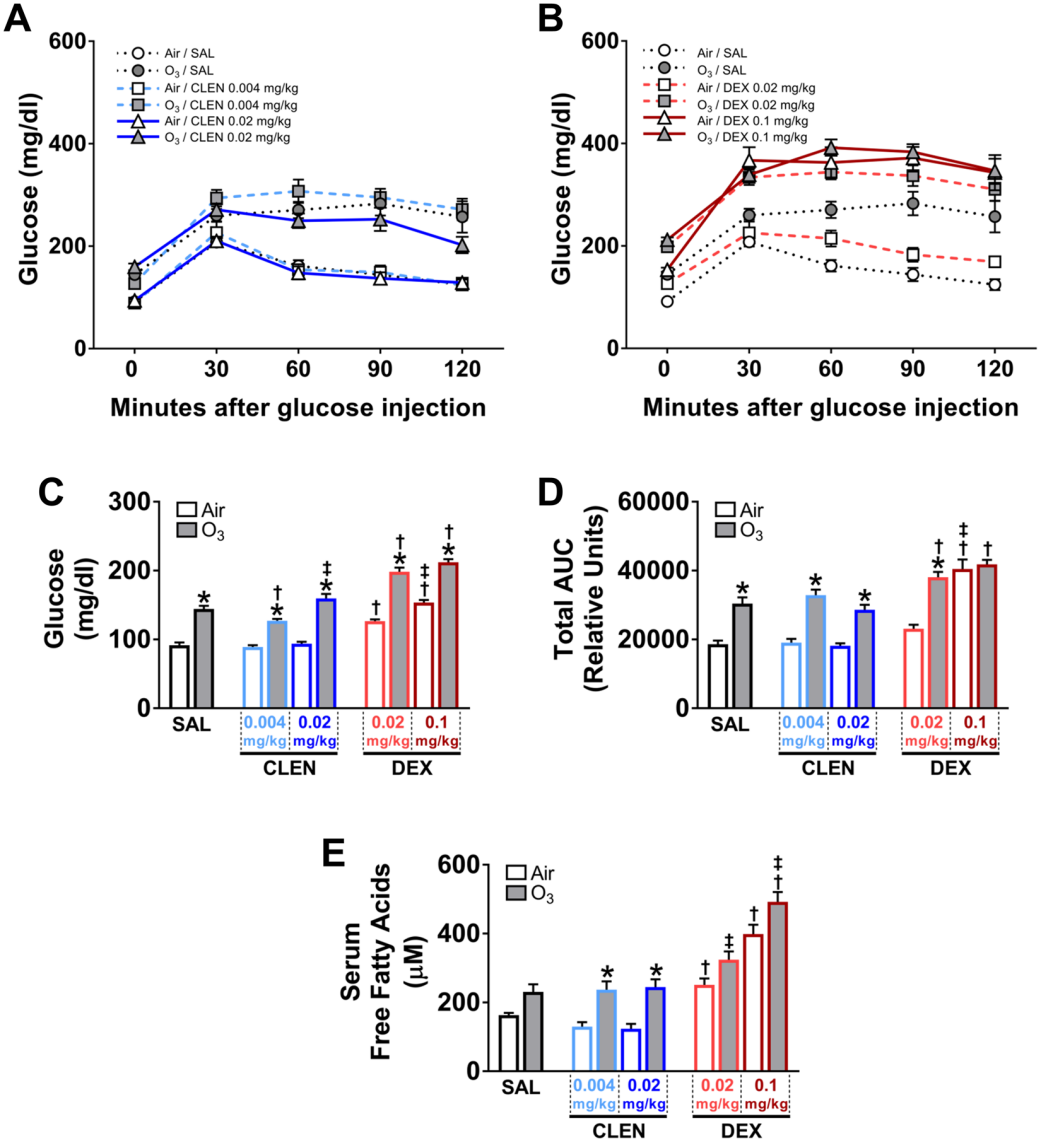
DEX and/or ozone exposure but not CLEN treatment induced hyperglycemia, glucose intolerance, and increased free fatty acids (study 1). Glucose tolerance test for CLEN treatment (**A**) and DEX treatment (**B**), baseline glucose levels before glucose tolerance test (**C**) and area under the curve (AUC) from glucose tolerance test (**D**) were obtained within 0.5–2.5 hr following the first day of air or ozone (0.8 ppm) exposure in rats (4 hr/day). Circulating free fatty acids were analyzed in serum samples collected during necropsy (**E**). Bar graphs show mean ± SEM of n = 6–8 animals/group. Significant differences between groups (p value ≤ 0.05) are indicated by * for ozone effect when compared to corresponding air-exposed rats, † for drug effect when compared to vehicle (SAL; saline)-treated rats, and ‡ for drug dose effect for the same exposure.

**Figure 9:**
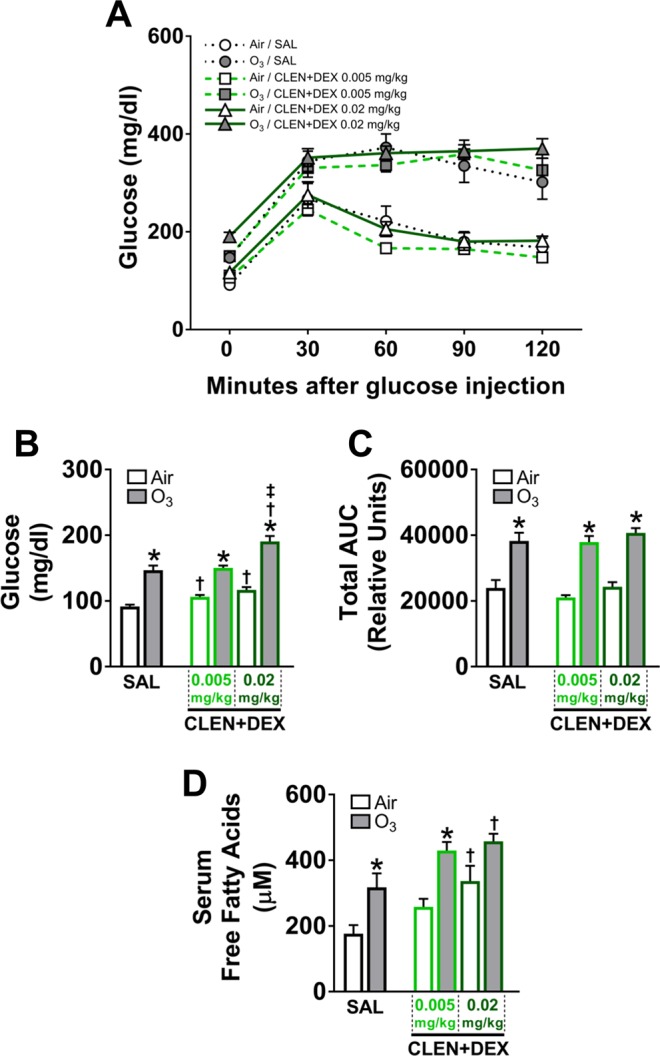
CLEN + DEX treatment alone increased blood glucose and circulating free fatty acids, while ozone exposure induced hyperglycemia glucose intolerance, and increased free fatty acids (study 2). Glucose tolerance test for CLEN + DEX treatment (**A**), baseline glucose levels before glucose tolerance test (**B**) and area under the curve (AUC) from glucose tolerance test (**C**) were obtained within 0.5–2.5 hr following the first day of air or ozone (0.8 ppm) exposure in rats (4 hr/day). Circulating free fatty acids were analyzed in serum samples collected during necropsy (**D**). Bar graphs show mean ± SEM of n = 6–8 animals/group. Significant differences between groups (p value ≤ 0.05) are indicated by * for ozone effect when compared to corresponding air-exposed rats, † for drug effect when compared to vehicle (SAL; saline)-treated rats, and ‡ for drug dose effect for the same exposure.

Our prior studies have shown that acute ozone exposure induces neuroendocrine stress-mediated systemic metabolic alterations^1,3^, and therefore, we examined the interactive influence of stress hormone receptor agonists CLEN and DEX on ozone-induced changes in glucose metabolism. Glucose tolerance testing was performed in air- and ozone-exposed rats to determine the modifying effects of CLEN (Fig. 8A) or DEX (Fig. 8B) in study 1 and CLEN + DEX (Fig. 9A) in study 2.

In study 1, DEX but not CLEN treatment produced hyperglycemia (baseline glucose levels at 0 min) and glucose intolerance in air-exposed rats (Fig. 8A–D). Ozone exposure also caused hyperglycemia and glucose intolerance in SAL-treated rats where DEX effect was further exacerbated by ozone exposure (both hyperglycemia and glucose intolerance) (Fig. 8A–D). Area under the curve assessment confirmed DEX effect and the exacerbation of the ozone effect on glucose intolerance in DEX-treated rats (Fig. 8D). Acute increase in circulating free fatty acids in response to ozone exposure was indicative of adipose lipolysis likely mediated by glucocorticoids^32^. Treatment with DEX at both doses significantly increased serum free fatty acids in air-exposed rats (Fig. 8E). Ozone tended to increase free fatty acids in SAL-treated rats. This effect of ozone was significant in CLEN-treated rats. Ozone-exposed DEX-treated rats had additive increases in circulating free fatty acids (Fig. 8E).

In study 2, high dose CLEN + DEX treatment produced hyperglycemia to a small degree but not glucose intolerance in air-exposed rats (Fig. 9B,C). Ozone exposure induced hyperglycemia and glucose intolerance in SAL-treated rats (Fig. 9A–C). Ozone-induced hyperglycemia (Fig. 9B) but not glucose intolerance response (Fig. 9C) was more pronounced in rats treated with high dose CLEN + DEX. Treatment with CLEN + DEX at the high dose significantly increased serum free fatty acids in air-exposed rats (Fig. 9D). Ozone exposure also increased free fatty acids levels in SAL and CLEN + DEX-treated rats (Fig. 9D).

### Stress hormone levels were variably altered by ozone exposure and drug treatment

Although ozone exposure has been shown to increase circulating epinephrine and corticosterone in some of our prior studies^1,3,6^, in the present study, the effects were inconsistent. Ozone exposure and drug treatments had mixed effects on circulating epinephrine and corticosterone. In study 1, the individual CLEN or DEX treatment did not change levels of epinephrine; however, high dose CLEN + DEX in study 2 significantly increased epinephrine in air-exposed rats (Table 1). Ozone exposure tended to increase epinephrine levels in SAL-treated rats. CLEN, DEX or CLEN + DEX had minimal effects on ozone-induced increases. Plasma levels of corticosterone were not affected by CLEN but were significantly decreased in all air and ozone-exposed rats treated with low and high doses of DEX in study 1 and high dose of CLEN + DEX in study 2. Ozone exposure in SAL rats did not significantly change corticosterone levels (Table 1).

**Table 1:** Plasma stress hormone levels in drug treated animals following acute air or ozone exposure.

	STUDY 1					
		SAL	CLEN LOW	CLEN HIGH	DEX LOW	DEX HIGH
Epinephrine	Air	59.4 ± 14.6	55.6 ± 27.8	35.3 ± 11	44 ± 9.8	46.9 ± 11.8
(pg/ml)	Ozone	87.8 ± 16.1	49.8 ± 7.1	58 ± 12.3	72.2 ± 10.8	84.7 ± 12.7
Corticosterone	Air	375.7 ± 77.2	368 ± 56.3	292.4 ± 80.5	18.8 ± 7.7^†^	10.2 ± 1^†^
(ng/ml)	Ozone	462.5 ± 48.6	278.7 ± 24.7	319.1 ± 27.1	134.1 ± 32.6*^†^	8.8 ± 0.5^†‡^
		**STUDY 2**				
		**SAL**	**CLEN** + **DEX** **LOW**	**CLEN** + **DEX** **HIGH**		
Epinephrine	Air	57 ± 6.3	47.5 ± 5.7	205.6 ± 160.5		
(pg/ml)	Ozone	142.3 ± 13	92.3 ± 13.9	79.7 ± 5.2		
Corticosterone	Air	580.5 ± 93.7	328.4 ± 92.4	36.7 ± 16.4^†‡^		
(ng/ml)	Ozone	384.4 ± 95.2	166.3 ± 43.7	99.3 ± 41.8^†^		

Values represent mean ± SEM of n = 6–8/group. Significant differences between groups (p value ≤ 0.05) are indicated by ^*^for ozone effect when compared to corresponding air-exposed rats, ^†^for drug effect when compared to corresponding vehicle (SAL)-treated rats, and ^‡^for drug dose effect for the same exposure. SAL – saline; CLEN – clenbuterol; DEX – dexamethasone.

## Discussion

Asthmatics receiving maintainance medication (such as LABA and/or glucocorticoids) experience exacerbated pulmonary injury and inflammation when exposed to high levels of air pollution^19,20^. We have recently reported that the combination treatment of CLEN + DEX at non-therapeutic high doses (0.2 mg/kg/day and 2 mg/kg/day respectively for 3 days) amplified ozone-induced pulmonary injury and inflammation in sham and adrenalectomized rats^8^. Building upon this, the goal of this study was to determine if β_2_AR and/or GR agonists when given at near therapeutic dose range, individually as singular therapy or as combination, (recommended by clinicians for asthma and COPD) would exacerbate acute ozone-induced lung injury/inflammation and also systemic metabolic alterations that were not reported in our previous study^8^.

In brief, we previously showed that ozone exposure in vehicle-treated rats caused lung injury, inflammation and BALF cytokine increases^6–8^. Similar to DEX treatment, ozone exposure also induced lymphopenia, decreased spleen weights and produced systemic metabolic alterations including hyperglycemia, glucose intolerance, and the release of free fatty acids into the circulation. Importantly, CLEN, even at the therapeutic concentrations used herein markedly exacerbated ozone-induced lung protein leakage and BALF cytokine release and neutrophilic influx seemingly, without exacerbating systemic effects of ozone. DEX, on the other hand, induced effects such as lymphopenia, hyperglycemia, glucose intolerance and free fatty acid release, that were similar to and more pronounced than those induced by ozone exposure alone. In general, the combination of CLEN + DEX at therapeutic levels showed less remarkable exacerbation of ozone pulmonary and systemic effects relative to CLEN alone. These data demonstrate that ozone-induced pulmonary injury and neutrophilic inflammation are exacerbated in rats receiving singular treatment of LABA (i.e. CLEN) at dose levels used, whereas, systemic immunological and metabolic alterations induced by ozone mimicked those of DEX with both producing additive effects. Thus, the pulmonary and systemic health effects of irritant air pollutants in those using LABA and steroids individually or as combination might be exacerbated because ozone inflammatory and metabolic effects are also mediated through the activation of AR and GR.

Ozone exposure has been shown to induce neuroendocrine-mediated release of adrenal-derived stress hormones, however because of the ultradian fluctuations^33,34^ and the influence of other stressors, often the increases in circulating hormones are not evident as we have seen in the present study. However, we have shown that the activation of genes responsive to AR and GR as well as lymphopenia, likely mediated by epinephrine and corticosterone release are readily apparent after ozone exposure^6,8,14^. It is noteworthy that, the treatment with DEX was associated with marked reduction in circulating corticosterone but not epinephrine levels. DEX, has been shown to reduce corticosterone levels due to negative feedback control on the hypothalamus and pituitary to activate CRH-mediated ACTH release^35,36^.

After ozone exposure, adrenal-derived stress hormones mediate their effects on homeostatic processes through AR and GR activation. In the present study, the exacerbation of ozone-induced pulmonary injury and inflammation in rats receiving therapeutic doses of CLEN or CLEN + DEX could be due to the high affinity of epinephrine for β_2_AR which represent ~80% of the total AR subtype in the airways and plays an important role in maintaining lung microvascular permeability, and airway smooth muscle as well as epithelial functions^37^. The activation of β_2_AR in airways has been shown to induce inflammatory changes associated with asthma phenotype in a mouse model^38^. We noted that ozone-induced pulmonary neutrophilia and increases in BALF IL-6 were exacerbated by CLEN or CLEN + DEX but not DEX. Although BALF TNF-α seem to show a small increase in CLEN-treated animals exposed to ozone, because of the inconsistency in ozone effects, and low level of this cytokine in BALF, its role in inflammatory response in current experimental setting is unclear. The lack of exacerbation or even slight dampening of ozone-induced neutrophilic inflammation and IL-6 release by DEX might be due to its immunosuppressive effects in the absence of CLEN. Thus, it is plausible that β_2_AR agonists use a similar mechanism as the endogenous epinephrine to exacerbate ozone-induced inflammation. The microvascular dilation induced by epinephrine^39^ or CLEN could facilitate this process by longer retention of blood in the lung and thus, facilitating neutrophil extravasation to the interstitial spaces in ozone-primed lungs. Immunomodulation of neutrophil release, recruitment and extravasation are dynamically controlled by both glucocorticoids and catecholamines^40,41^.

Acute ozone exposure has been shown to induce ventilatory changes in sensitive rat strains such as Wistar Kyoto rats^42^ resulting in increased Penh^8^, which has been correlated with labored breathing^31^. We previously showed that a non-therapeutic high dose CLEN + DEX treatment resulted in minimal changes in ventilatory parameters when administered to air-exposed rats, however in ozone-exposed rats there were marked increases in pulmonary edema and corresponding ventilatory changes^8^. At the lower dosages of CLEN or CLEN + DEX used in the present study, we also did not observe significant changes in ventilatory parameters in air-treated rats. However, despite using more clinically relevant dosages of CLEN, DEX or the combined treatment, we still were not able to demonstrate improvement in the ozone-induced ventilatory changes. Results suggest that the airflow obstruction and lung injury/pulmonary edema induced by ozone are resistant to glucocorticoid or β_2_AR agonists treatment.

Although ozone has been shown to induce pulmonary protein leakage in many studies, the precise contributions of hemodynamic and non-hemodynamic mechanisms are unclear. Our previous study showed that a high dose of CLEN plus DEX even without ozone caused lung protein leakage^8^ suggesting the contribution of βAR and circulating epinephrine. This is supported by the evidence that in the presence of therapeutic levels of CLEN, which did not produce edema in air-exposed rats, ozone-induced pulmonary vascular leakage was markedly exacerbated. In the adrenalectomized rats ozone-induced vascular leakage was minimal^5^. Epinephrine-mediated activation of βAR has been implicated in increased lung permeability^43,44^. Since, the predominant response in the lung of β_2_AR activation is likely to be vasodilation and bronchodilation, the increases in epinephrine together with CLEN and hemodynamic changes associated with ozone-induced bradycardia might promote fluid filling in the microvasculature^45,46^. When DEX is combined with CLEN (CLEN + DEX) exacerbation of ozone-induced lung protein leakage is less dramatic than that occuring in rats with CLEN-only treatment, suggesting that GR and β_2_AR might interactively maintain homeostatic processes to reduce injury induced by a stressor. It may be relevant that, increased risk of asthma-related death by LABA monotherapy is not evident in asthmatic patients taking a combination of LABA plus corticosteroids^12,47^.

The neuroendocrine response induced by ozone exposure is associated with the changes in systemic immunological surveillance^48^. Increased numbers of activated macrophages have been found in the lung after ozone exposure^49,50^. Moreover, splenic macrophages have been shown to migrate to the lungs after ozone inhalation^51^. In this study, CLEN-alone treatment increased the number of BALF macrophages in ozone-exposed rats, suggesting that it likely affected macrophage extravasation. Macrophages express β_2_AR and can be activated by LABA treatment^52^.

DEX at the concentrations used in both studies induced lymphopenia and reduced thymus and spleen weights, demonstrating the immunosuppressant property. Similar to DEX, ozone exposure has previously shown to induce lymphopenia^6,7,53^ and thymic involution^54–56^. In this study, we noted lymphopenia as well as small reductions in thymus and spleen weights after acute ozone exposure; effects that were clear in rats treated with DEX or CLEN + DEX. Concomitantly, GR blockade but not βAR inhibition, prevented ozone-induced lymphopenia^7^, suggesting that endogenous GR signaling was required. Both spleen and thymus T lymphocytes were decreased in ozone exposed mice^54^. Collectively, these data suggest that AR and GR, which are therapeutically modulated could affect ozone-induced pulmonary innate immune changes.

In addition to immune surveillance, the stress-induced neuroendocrine activation also orchestrates metabolic energy redistribution and conservation for future needs^57^. Ozone exposure has been shown to induce a shift in metabolites distribution in multiple organs in rats and in humans^1,3,5^. In this study, although the use of β_2_AR specific agonist CLEN was targeted to understand its role in the lung tissue, we presumed that CLEN would also have systemic effects, since these receptors are distributed in vascular smooth muscle involved in vasodilation. However, we noted that DEX but not CLEN, independently induced hyperglycemia, glucose intolerance and lipolysis as evident by free fatty acids release. The metabolic changes in CLEN + DEX-treated rats with or without ozone exposure were indicative of the predominant role of glucocorticoids. The understanding of how chronic stimulation of these metabolic processes following long-term pollutant exposure lead to metabolic diseases is critical for causal evidence to support epidemiological associations^58,59^.

Some limitations are noted in this study. First, both CLEN and DEX were delivered intraperitoneally to precisely control the dose and to assess pulmonary as well as systemic effects. However, direct lung delivery, which is often used for asthma and COPD treatment, might result in different outcomes. The effectiveness of pulmonary delivery needs to be examined in future studies. Second, the rats are not sensitized or “asthmatic”, while people receiving β_2_AR agonists are primarily asthmatic individuals. Since asthmatic individuals are more sensitive to ozone-induced inflammation^19,20,60^, it is possible that the exacerbation resulting from β_2_AR agonist usage during air pollution exposure might be of even greater magnitude. The interactive effect of CLEN, DEX and ozone will need to be examined in an animal model of allergic asthma.

In conclusion, CLEN treatment at therapeutically-relevant dosages, exacerbated ozone-induced pulmonary injury/ inflammation and BALF inflammatory cytokine release through the activation of β_2_AR, likely the same mechanism by which ozone also induces vascular leakage and inflammation. The degree of CLEN-related exacerbation of ozone-induced pulmonary effects was reduced when rats were treated with CLEN + DEX combination, while DEX by itself did not influence this pulmonary outcome. However, even at lower than therapeutic dose levels, DEX treatment was associated with systemic metabolic and immunological effects in air-exposed rats that were similar to effects induced by ozone and often additive, suggesting a similar mode of action through the activation of GR. Clinically, the effect of LABA on β_2_AR is important as inner-city children, who are more prone to asthma and use bronchodilators, are likely to be exposed to high levels of air pollution^61,62^. These potential drug-air pollution interactions might be critical in those socioeconomically disadvantaged communities with disproportional exposure to elevated levels of air pollution, where high incidence of chronic lung and metabolic diseases coincide with the use of bronchodilators and/or steroids.
